# Identification and evolution of C_4_ photosynthetic pathway genes in plants

**DOI:** 10.1186/s12870-020-02339-x

**Published:** 2020-03-30

**Authors:** Weiping Shi, Linqi Yue, Jiahui Guo, Jianming Wang, Xiangyang Yuan, Shuqi Dong, Jie Guo, Pingyi Guo

**Affiliations:** College of Agronomy, Shanxi Agricultural University, Taigu, 030801 China

**Keywords:** C_4_, Evolution, *NADP-ME*, *PPDK*, Positively selected sites

## Abstract

**Background:**

NADP-malic enzyme (NAPD-ME), and pyruvate orthophosphate dikinase (PPDK) are important enzymes that participate in C_4_ photosynthesis. However, the evolutionary history and forces driving evolution of these genes in C_4_ plants are not completely understood.

**Results:**

We identified 162 *NADP-ME* and 35 *PPDK* genes in 25 species and constructed respective phylogenetic trees. We classified *NADP-ME* genes into four branches, A1, A2, B1 and B2, whereas *PPDK* was classified into two branches in which monocots were in branch I and dicots were in branch II. Analyses of selective pressure on the *NAPD-ME* and *PPDK* gene families identified four positively selected sites, including 94H and 196H in the a5 branch of NADP-ME, and 95A and 559E in the e branch of PPDK at posterior probability thresholds of 95%. The positively selected sites were located in the helix and sheet regions. Quantitative RT-PCR (qRT-PCR) analyses revealed that expression levels of 6 *NADP-ME* and 2 *PPDK* genes from foxtail millet were up-regulated after exposure to light.

**Conclusion:**

This study revealed that positively selected sites of NADP-ME and PPDK evolution in C_4_ plants. It provides information on the classification and positive selection of plant *NADP-ME* and *PPDK* genes, and the results should be useful in further research on the evolutionary history of C_4_ plants.

## Background

Photosynthesis is the process used by plants to convert solar energy into chemical energy. This enables them to produce their own food for development [1]. Photosynthesis in higher plants can be classified into C_3_, C_4_ and Crassulacean acid metabolism (CAM) based on how they fix carbon during the process that leads to different initial photosynthesized products. The majority of land plants use the C_3_ pathway, whereas C_4_ and CAM plants were evolved from C_3_ plants [2, 3]. C_4_ plants are more efficient than C_3_ plants in utilizing CO_2_ leading to superior adaptiveness to subtropical and tropical environments, lower concentrations of CO_2_, and more stressed environments [4]. Numerous studies have focused on understanding the efficiency and the mechanism of carbon fixation in C_4_ plants [5, 6].

Among the many enzymes involved in the C_4_ photosynthesis pathway PPDK and NADP-ME are considered to be the most important [7, 8].

PPDK is a critical enzyme that controls the photosynthetic rate in C_4_ plants [9]. Many *PPDK* genes in C_4_ and CAM plants have been cloned, exemplified by those in maize and *Mesembryanthemum crystallinum* [10, 11]. A phylogenetic study suggested that *PPDK* genes in sorghum and rice are homologous [12]. Detailed analysis of *PPDK* isoform sequences between the Poaceae and Arabidopsis indicated that their sequences share about 20 amino acids of chloroplast transit peptide (cTP), proving that the *PPDK* genes had evolved before divergence of monocots and dicots [12].

*NADP-ME* genes can be classified into photosynthetic and non-photosynthetic types. The former mostly function in the chloroplasts [13] and improve photosynthetic efficiency by facilitating the release of CO_2_ from decarboxylation of malate in proximal bundle-sheath cells, and in C_4_ plants by providing CO_2_ to Rubisco for carbon fixation [14, 15]. Genomic and phylogenetic analyses showed that the *NADP-ME* gene family in the Poaceae has four branches, with one branch (*NADP-ME* IV) being expressed in the plastids. The C_4_-specific *NADP-ME* has some codons suppressed under positive selection and is independent of the *NADP-ME* IV family [16, 17].

Natural selection, a key factor in biological evolution, includes positive selection, purifying selection, and neutral selection [18]. The base substitution rate (non-synonymous/synonymous, ω = dN/dS), an index that determines selection pressure after change, is typically used to understand the direction of evolution and its selective strength in a coding sequence. If ω > 1, a gene might undergo positive selection or presence of a new amino acid offers a fitness advantage; ω =1 is indicative of neutral selection; and a value of ω < 1 indicates purifying selection [19]. As an important basis of adaptive evolution, positive selection functions in a population by favorable transmission and increased frequency of a mutant allele [18].

Positive selection often implies the emergence of a new function [19, 20]. In transformation of the C_3_ to C_4_ pathway positive selection mainly occurred in key enzymes in C_4_ photosynthetsis, such as Rubisco, phosphoenolpyruvate carboxylase (PEPC), NADP-ME, and PPDK [12, 21–26]. For example, two positively selected large subunit (LSu) amino acid substitutions, M309I and D149A, distinguish C_4_ Rubiscos from the ancestral C_3_ species [21]. With the switch to C_4_, 21 amino acids evolved under positive selection and converged to similar or identical amino acids in most of the grass C_4_ PEPC lineages [22]. Acquisitions of C_4_ PEPC in sedges (*Cyperaceae*) were driven by positive selection on at least 16 codons [23]. Previous studies used variation in amino acids to study rates of evolution in the C_4_-NADP-ME pathway, and a number of residues was found to be under significant positive selection [24]. During independent evolution of NADP-ME in C_4_ plants strong positive selection led to sequence convergence [25]. For example, among the 29 residues of C_4_ NADP-MEs and non C_4_ NADP-MEs, residues 284, 450 and 539 were identified as having been under positive selection during evolution of C_4_-NADP-ME in grasses, suggesting they were important in explaining kinetic and structural differences between C_4_ and non-C_4_ groups [26]. Phylogenetic analysis also suggested that the maize *PPDK* gene and its sorghum ortholog were under significant positive selection, implying possible functional changes [12].

The underlying molecular mechanisms of C_4_ photosynthesis are poorly understood and few studies have been directed to understanding whether positive selection was associated with evolution of NADP-ME and PPDK in C_4_ plants. Completion of the whole genome sequences of C_4_ plants such as sorghum and maize [27, 28], and improved knowledge of photosynthetic pathways and evolution, have set a solid foundation for study of the evolution and expression of key C_4_ enzyme genes. A comparison of the *PPDK* and *NADP-ME* gene families in C_4_ plants could advance knowledge of the evolutionary, functional and metabolic roles of these genes during photosynthesis. This study investigated the evolutionary processes in *NADP-ME* and *PPDK* in algal, moss, Lycopodiophyta, monocotyledon and dicotyledon species, providing new information regarding C_4_ photosynthesis.

## Results

### Numbers of *NADP-ME* and *PPDK* genes in plants

A total 162 NADP-ME and 35 PPDK sequences were found in 25 species, including one algal, one moss, one Lycopodiophyta, 10 monocot (including 6 C_4_), and 12 dicot (including 1 C_4_) species (Additional file 1: Table S1; Additional file 2: Table S2). There were 14 *NADP-ME* genes in soybean. Carrot, cotton and poplar each had 9 *NADP-ME* genes and *Selaginella moellendorffii* had 3 (Additional file 1: Table S1). The number of *PPDK* genes was far fewer, with the largest number being 3 in the banana species *Musa acuminata*. Most other species had only 1 or 2 *PPDK* genes (Additional file 2: Table S2).

### Analysis of conserved amino acid sequences in NADP-ME and PPDK proteins

The MEME program used to analyze conserved sequences in NADP-ME and PPDK proteins identified 20 motifs (Additional file 3: Table S3; Additional file 4: Table S4). Among *NADP-ME* genes, those from algae (*Cre14.g629700.t1.1*, *Cre14.g628650.t1.2*, *Cre14.g629750.t2.1*, *Cre01.g022500.t1.2*) did not contain motifs 13, 14, 15, 17 and 19. Subfamily A had two unique motifs, 17 and 19, whereas subfamily B had three unique motifs, 13, 14 and 15 (Additional file 5: Figure S1). The *PPDK* gene in green algae lacked motif 15, whereas all other *PPDK* genes had all 20 candidate motifs (Additional file 6: Figure S2).

### Phylogeny of *NADP-ME* and *PPDK*

**Fig. 1:**
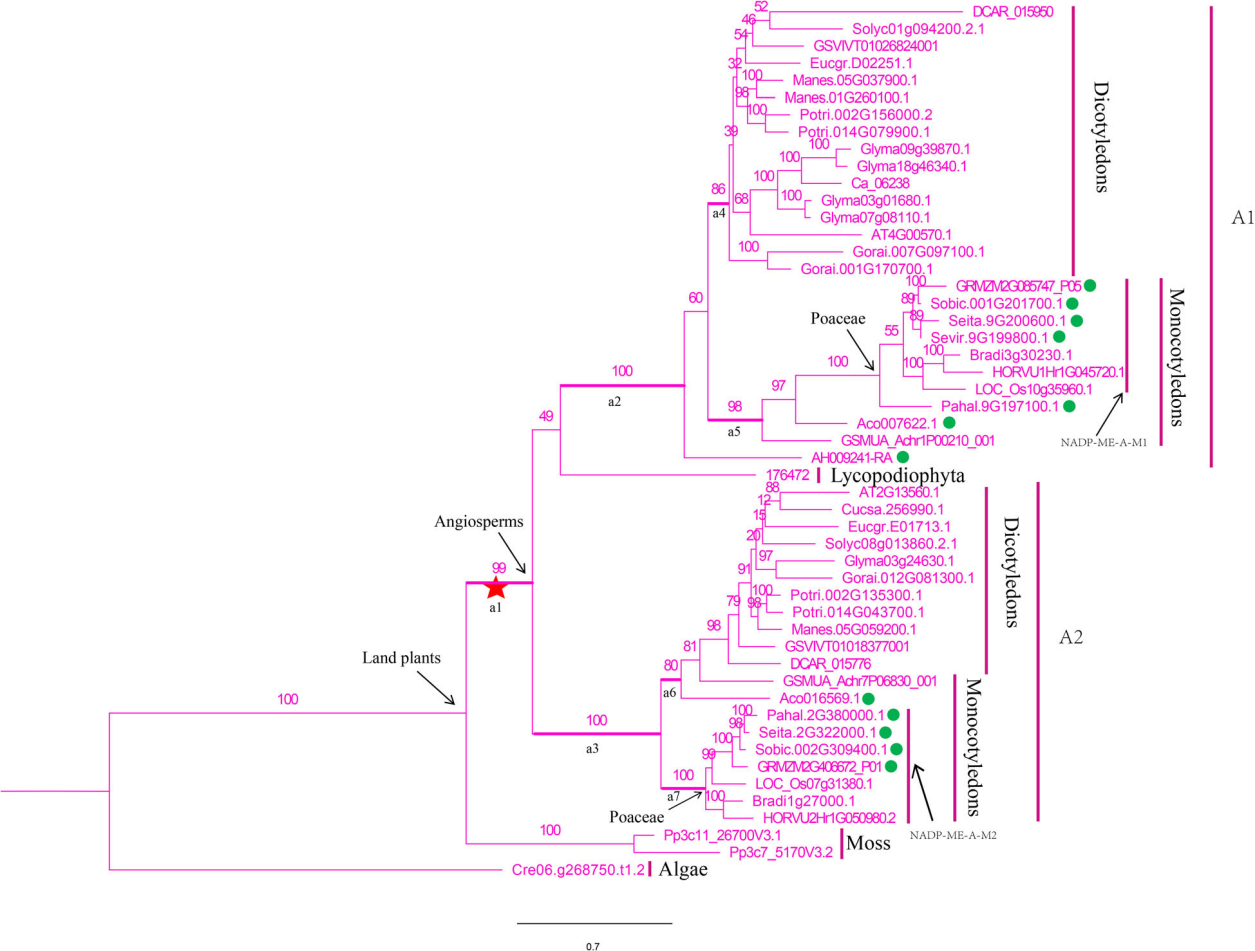
Phylogenetic tree established for the *NADP-ME* gene family in 25 species (A branch). The A branch was further diverged into subfamilies A1 and A2. For the branch model, a1-a7 were assigned to be the front branches in the selective pressure analysis. C_4_ plants are marked with green circles

**Fig. 2:**
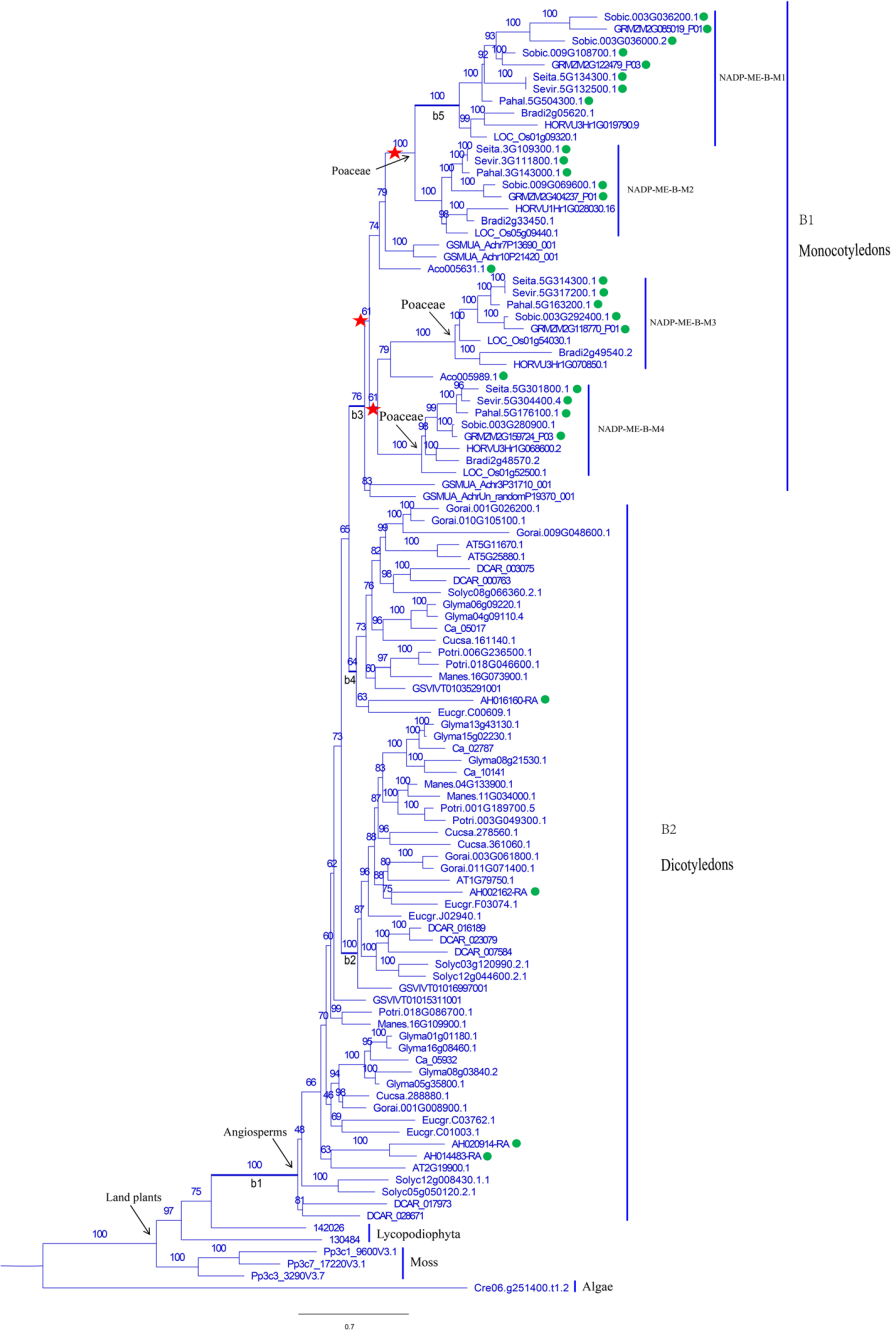
Phylogenetic tree established for the *NADP-ME* gene family in 25 species (B branch). The B branch was further diverged into subfamilies B1 and B2. For the branch model, b1-b5 were assigned as front branches in the selective pressure analysis. C_4_ plants are marked with green circles

We constructed a phylogenetic tree for all 162 *NADP-ME* genes from 25 species and discovered that they shared a common ancestor. The algal *NADP-ME* was the most ancient gene and was divergent from the rest of the clade. Subfamilies A and B separated after whole genome duplication (Additional file 7: Figure S3). In subfamily A, the *NADP-ME* gene in algae branched off first, and the rest were classified into subfamilies A1 and A2. A clear clustering between monocot and dicot plants for each subfamily was observed. Among the A1 and A2 monocot branches, *NADP-ME* in *Musa acuminata* and *Ananas comosus* branched off before the Poaceae. Within the Poaceae, *NADP-ME* genes in C_4_ plants were more closely related to each other (Fig. 1). In the B subfamily, the *NADP-ME* genes of algae again branched off first, followed by the land plants *Physcomitrella patens* and *Selaginella moellendorffii*. Among angiosperm species, *NADP-ME* from dicots (B2 subfamily) branched first and *NADP-ME* in the monocots diverged after gene duplication and formed the B1 subfamily which underwent three whole genome duplication events. Like the A subfamily, the *NADP-ME* in *Musa acuminata* and *Ananas comosus* branched off earlier than counterpart in the Poaceae in which there were four branches, namely, NADP-ME-B-M1, NADP-ME-B-M2, NADP-ME-B-M3 and NADP-ME-B-M4 (Fig. 2). We discovered that the *NADP-ME* genes were clustered and closely related within each of the C_3_ and C_4_ species groups.

**Fig. 3:**
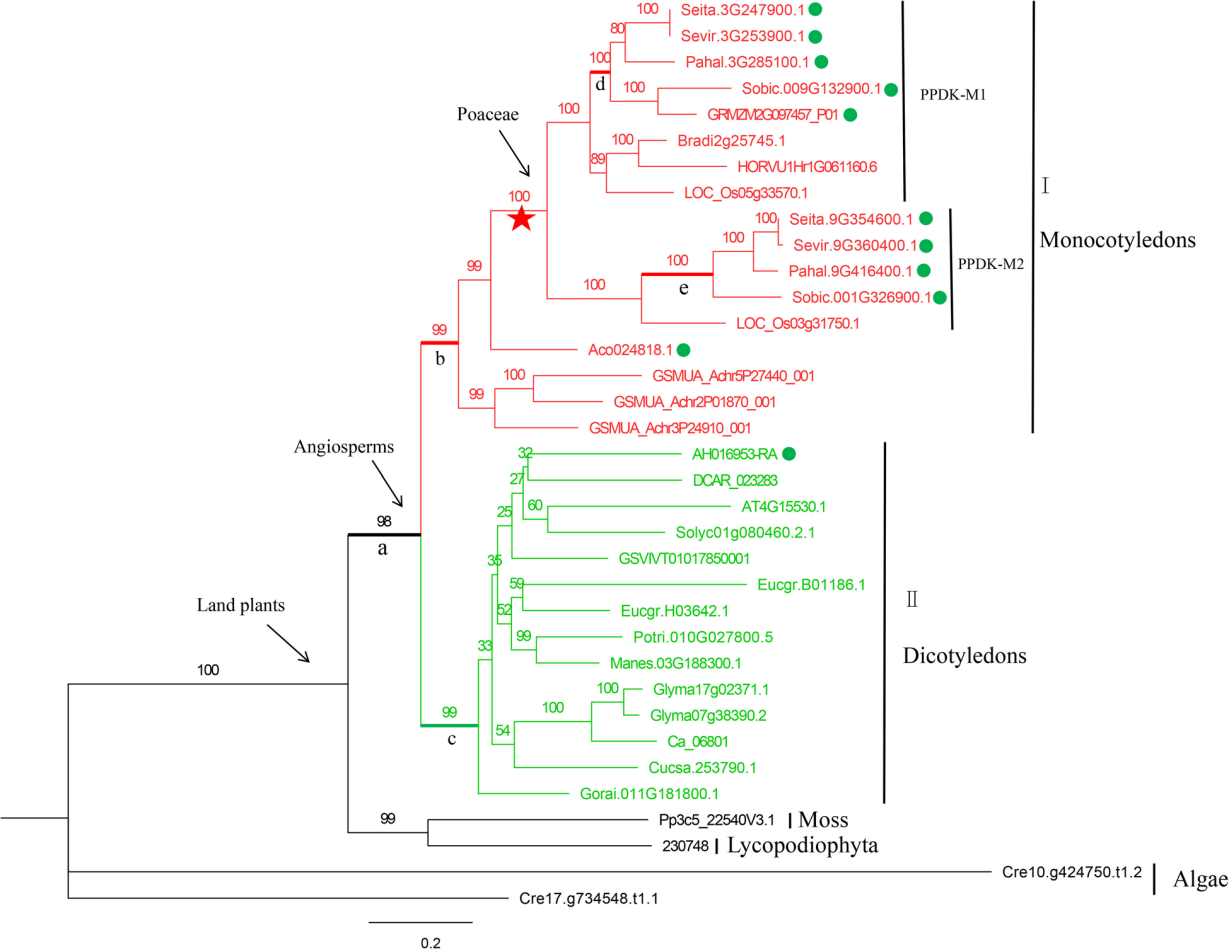
Phylogenetic tree established for *PPDK* gene family in 25 species, including branches I and II. For the branch model, a-e were assigned as front branches in the selective pressure analysis. C_4_ plants are marked with green circles

All 35 *PPDK* genes from 25 species were used to construct a phylogenetic tree. The *PPDK* gene in green algae was first to branch off, and there was further divergence into subfamilies I and II. Subfamily I consisted of monocots and subfamily II consisted of dicots. The *PPDK* gene in subfamily I first appeared in *Musa acuminata* and *Ananas comosus* and later diverged to the Poaceae. Whole genome duplication then occurred after this divergence, and two main branches were formed, with one branch including barley, maize and *Brachypodium distachyon* showing loss of the *PPDK* gene or lack of a conserved *PPDK* structure. It was also discovered that *PPDK* genes in C_4_ plants are closely related (Fig. 3).

### Analysis of selection pressure on *NADP-ME* and *PPDK* genes

Selection pressures within each of the A and B subfamilies of *NADP-ME* genes were investigated. In the subfamily A, the M0 and M3 models were based on the site model for calculation. Under the M0 model ω was 0.091, indicating that it was under purifying selection. The *P*-value from the chi-squared test comparing the M0 and M3 models was 0.000, suggesting that the ω value were not constant across loci (Table 1). For the branch model, seven branches, a1-a7, were assigned as front branches. The branch model results showed that the ω values for all front branches were < 1. Likelihood ratio tests (LRT) showed that branches a1, a2 and a3 were significantly different from the other branches with all ω values < 1 thus suggesting purifying selection (Fig. 1; Table 1). The branch-site model revealed that the proportions of positive selection at a1-a5 were 5, 0.2, 5.8, 11 and 1.5%, respectively, whereas the proportions at a6 and a7 were close to 0. The numbers of positively selected sites for a1-a7 were 8, 5, 14, 5, 4, 2 and 2 at a posterior probability of 0.6. The LRT result suggested that branches a1, a3 and a5 were significantly different from the M1 model (*P* <  0.05). Interestingly, the a1 branch, ancestral to subfamilies A1 and A2, was stabilized after positive selection at both the a1 and a3 branches. On the contrary, a4 and a5 still had positively selected sites following positive selection at the a2 branch. This suggested that subfamily A1 had undergone different levels of positive selection at different branches. The a5 and a7 branches comprised mostly monocots and C_4_ plants (Fig. 1; Table 1). For subfamily B, the ω values were similar to those of subfamily A on the site model. LRT indicated that subfamily B was still under purifying selection with ω values varying among sites (Table 2). Branches b1-b5, were under strong purifying selection with proportions of positively selected sites of 5.5, 0, 0.6, 8.9 and 1.8% and ω values much smaller than 1 based on the branch and branch-site models (Table 2). The numbers of positively selected sites for b1-b5 were 8, 0, 2, 0 and 5 at a posterior probability of 0.6 (Fig. 2; Table 2). It was concluded that b1 is the most ancient branch of *NADP-ME* genes in subfamily B with a total of 8 positively selected sites. The b3 and b5 branches had 2 and 5 positively selected sites, whereas both the b2 and b4 branches comprised dicots, with no positively selected sites at a posterior probability of 0.6, thus indicating that the b2 and b4 branches were more conserved than the b3 and b5 branches and that the evolutionary steps from b1 to b3 and b5 in Subfamily B were rather complex (Fig. 2; Table 2).

**Table 1:** Parameters in the site analysis, branch and branch-site analyses of *NADP-ME*-A

Test Used	Foregroud branches	Model	ω	LnL	Estimates of parameters	df	*P*	Positively selected sites under BEB
Site analysis	–	M0:(one-ratio)	0.091	−27,294.09	ω = 0.091	–	–	–
	–	M3:(discrete)	0.117	−26,141.38	p0 = 0.601 ω0 = 0.019	4	0.000(M0 vs M3)	–
					p1 = 0.360 ω1 = 0.175			
					p2 = 0.039 ω2 = 1.091			
Branch analysis	–	M0	–	−27,294.09	ω = 0.091	–	–	–
	–	a1	–	−27,286.99	ω_0_ = 0.092 ω_1_ = 0.006	1	< 0.001	–
	–	a2	–	−27,287.52	ω_0_ = 0.092 ω_1_ = 0.016	1	< 0.001	–
	–	a3	–	−27,285.91	ω_0_ = 0.092 ω_1_ = 0.018	1	< 0.001	–
	–	a4	–	−27,292.69	ω_0_ = 0.090 ω_1_ = 0.314	1	0.095	–
	–	a5	–	−27,294.06	ω_0_ = 0.091 ω_1_ = 0.085	1	0.827	–
	–	a6	–	−27,293.33	ω_0_ = 0.090 ω_1_ = 0.358	1	0.218	–
	–	a7	–	−27,292.88	ω_0_ = 0.090 ω_1_ = 0.158	1	0.121	–
Branch-site analysis	–	M1	–	−26,625.07	p_0_ = 0.915 p_1_ = 0.085	–	–	–
	a1	Model A	–	−26,616.55	p0 = 0.871 p1 = 0.079(p_2a_ + p_2b_ = 0.050)	2	0.0002 (ModelA vs M1)	Prob > 0.6 (178 M, 188Q, 193D, 326E, 330 L, 344 V, 345S, 375 L)
					ω2 = 37.078			
	a2	Model A	–	−26,622.12	p_0_ = 0.897 p_1_ = 0.081 (p_2a_ + p_2b_ = 0.002)	2	0.0520 (ModelA vs M1)	Prob > 0.6 (11 L, 63D^a^, 94H, 110R, 344 V)
					ω2 = 8.513			
	a3	Model A	–	−26,610.43	p_0_ = 0.861 p_1_ = 0.080 (p_2a_ + p_2b_ = 0.058)	2	< 0.0001 (ModelA vs M1)	Prob > 0.6 (11 L, 54R^a^, 66Q^b^, 180 M^a^, 188Q, 200A^a^, 248P, 273R^b^, 274I^a^, 275 T, 298 V, 344 V, 367 K, 389S)
					ω_2_ = 152.141			
	a4	Model A	–	−26,624.28	p_0_ = 0.801 p_1_ = 0.074 (p_2a_ + p_2b_ = 0.11)	2	0.4525 (ModelA vs M1)	Prob > 0.6 (122 V, 174A, 185 M, 220R, 268A)
					ω2 = 1.000			
	a5	Model A	–	−26,619.18	p_0_ = 0.902 p_1_ = 0.083 (p_2a_ + p_2b_ = 0.015)	2	0.0028 (ModelA vs M1)	Prob > 0.6(94H^a^, 182A, 196H^a^, 269E)
					ω2 = 11.724			
	a6	Model A	–	−26,625.07	p_0_ = 0.915 p_1_ = 0.085 (p_2a_ + p_2b_ = 0.000)	2	1.0000 (ModelA vs M1)	Prob > 0.6 (13H, 201H)
					ω2 = 3.081			
	a7	Model A	–	−26,625.07	p_0_ = 0.915 p_1_ = 0.085 (p_2a_ + p_2b_ = 0.000)	2	1.0000 (ModelA vs M1)	Prob > 0.6 (33 K, 56 L)
					ω2 = 1.000			

^a^Posterior probability > 95%

^b^Posterior probability > 99%

**Table 2:** Parameters in the site analysis, branch and branch-site analyses of *NADP-ME*-B

Test Used	Foregroud branches	Model	ω	LnL	Estimates of parameters	df	*P*	Positively selected sites under BEB
Site analysis	–	M0:(one-ratio)	0.081	−30,030.68	ω = 0.081	–	–	–
	–	M3:(discrete)	0.088	−29,088.89	p_0_ = 0.468 ω_0_ = 0.012	4	0.000 (M0 vs M3)	–
					p_1_ = 0.415 ω_1_ = 0.091			
					p_2_ = 0.117 ω_2_ = 0.376			
Branch analysis	–	M0	–	−30,030.68	ω = 0.081	–	–	–
	–	b1	–	−30,028.18	ω0 = 0.081 ω_1_ = 0.016	1	0.0254	–
	–	b2	–	−30,030.66	ω_0_ = 0.081 ω_1_ = 0.072	1	0.8415	–
	–	b3	–	−30,028.75	ω_0_ = 0.081 ω_1_ = 0.031	1	0.0495	–
	–	b4	–	−30,030.66	ω_0_ = 0.081 ω_1_ = 0.102	1	0.858	–
	–	b5	–	−30,030.46	ω_0_ = 0.081 ω_1_ = 0.112	1	0.5052	–
Branch-site analysis	–	M1	–	−29,644.79	p0 = 0.912 p1 = 0.088	–	–	–
	b1	Model A	–	− 29,633.47	p_0_ = 0.862 p_1_ = 0.083 (p_2a_ + p_2b_ = 0.055)	2	< 0.0001 (ModelA vs M1)	Prob > 0.6 (28R^a^, 59 L^a^, 108 V^a^, 125R, 144 T, 169 L, 181S^a^, 204 L)
					ω_2_ = 37.343			
	b2	Model A	–	−29,644.79	p_0_ = 0.912 p_1_ = 0.088 (p_2a_ + p_2b_ = 0.000)	2	1.0000 (ModelA vs M1)	Prob > 0.6 (NO)
					ω_2_ = 1.000			
	b3	Model A	–	−29,642.93	p_0_ = 0.906 p_1_ = 0.088 (p_2a_ + p_2b_ = 0.006)	2	0.1564 (ModelA vs M1)	Prob > 0.6 (59 L^a^, 182 L)
					ω2 = 19.072			
	b4	Model A	–	−29,645.74	p_0_ = 0.831 p_1_ = 0.080 (p_2a_ + p_2b_ = 0.089)	2	0.3852 (ModelA vs M1)	Prob > 0.6 (NO)
					ω2 = 3.081			
	b5	Model A	–	−29,641.83	p0 = 0.895 p1 = 0.0.087 (p2a + p2b = 0.018)	2	0.0518 (ModelA vs M1)	Prob > 0.6 (59 L, 63S, 86E^a^, 108 V, 181S)
					ω_2_ = 11.224			

^a^Posterior probability > 95%

For the *PPDK* gene family, the M0 and M3 models compared by LRT yielded a *P*-value of 0.000 based on the site model. This indicated that the ω values were not constant across sites, similar to the *NADP-ME* gene family results (Table 3). Branches a-e were assigned as foreground branches in the branch model, with their ω values much smaller than 1, suggesting purifying selection. Interestingly, the ω values from a to e were gradually increasing, with a (0.0006) < b (0.024) < c (0.026) < d (0.078) < e (0.284). This trend suggests that *PPDK* genes were under strong purifying selection in lower plants prior to divergence of monocots and dicots. Even after divergence of monocots and dicots from lower plants there was duplication of *PPDK* genes. The ancestral branch of both dicots and monocots (c, b, d) are still under strong purifying selection. Purifying selection on branch e, which contains C_4_ plants was declining (Fig. 3; Table 3). The branch-site model showed that the proportion of positively selected sites of branches a-d was close to 0, but in the case of branch e it was 4.4%. The numbers of positively selected sites of a-e were 1, 1, 0, 0, and 8 at a posterior probability of 0.6. Positively selected sites on branch e were statistically more than on the other four branches with *P* <  0.0001 (Fig. 3; Table 3).

**Table 3:** Parameters in the site analysis, branch and branch-site analyses of *PPDK*

Test Used	Foregroud branches	Model	ω	LnL	Estimates of parameters	df	*P*	Positively selected sites under BEB
Site analysis	–	M0:(one-ratio)	0.1	−34,454.01	ω = 0.095	–	–	–
	–	M3:(discrete)	0.11	−32,794.04	p_0_ = 0.562 ω_0_ = 0.009	4	0.000 (M0 vs M3)	–
					p_1_ = 0.302 _1_ = 0.117			
					p_2_ = 0.136 ω_2_ = 0.521			
Branch analysis	–	M0	–	−34,454.01	ω = 0.095	–	–	–
	–	a	–	− 34,433.04	ω0 = 0.0979 ω_1_ = 0.0006	1	< 0.0001	–
	–	b	–	−34,444.44	ω_0_ = 0.097 ω_1_ = 0.024	1	< 0.0001	–
	–	c	–	−34,441.56	ω0 = 0.098 ω_1_ = 0.026	1	< 0.0001	–
	–	d	–	−34,453.87	ω_0_ = 0.095 ω_1_ = 0.078	1	0.606	–
	–	e	–	−34,443.74	ω_0_ = 0.093 ω_1_ = 0.284	1	< 0.0001	–
Branch-site analysis	–	M1	–	−33,410.98	p_0_ = 0.844 p_1_ = 0.156	–	–	–
	a	Model A	–	−33,410.98	p0 = 0.844 p1 = 0.156(p_2a_ + p_2b_ = 0.000)	2	1.0000 (ModelA vs M1)	Prob > 0.6 (257I)
					ω2 = 1.000			
	b	Model A	–	−33,410.98	p_0_ = 0.844 p_1_ = 0.156 (p_2a_ + p_2b_ = 0.000)	2	1.0000 (ModelA vs M1)	Prob > 0.6 (337R)
					ω2 = 1.000			
	c	Model A	–	− 33,410.98	p_0_ = 0.844 p_1_ = 0.156 (p_2a_ + p_2b_ = 0.000)	2	1.0000 (ModelA vs M1)	Prob > 0.6 (NO)
					ω_2_ = 1.000			
	d	Model A	–	−33,410.98	p_0_ = 0.844 p_1_ = 0.156 (p_2a_ + p_2b_ = 0.000)	2	1.0000 ModelA vs M1)	Prob > 0.6 (NO)
					ω2 = 1.000			
	e	Model A	–	−33,400.15	p_0_ = 0.810 p_1_ = 0.147 (p_2a_ + p_2b_ = 0.044)	2	< 0.0001 (ModelA vs M1)	Prob > 0.6 (24 N, 95A^a^, 174I, 359A, 459G, 489A, 559E^b^, 561G)
					ω_2_ = 3.507			

^a^Posterior probability > 95%

^b^Posterior probability > 99%

### Protein structural characteristics of NADP-ME and PPDK

**Fig. 4:**
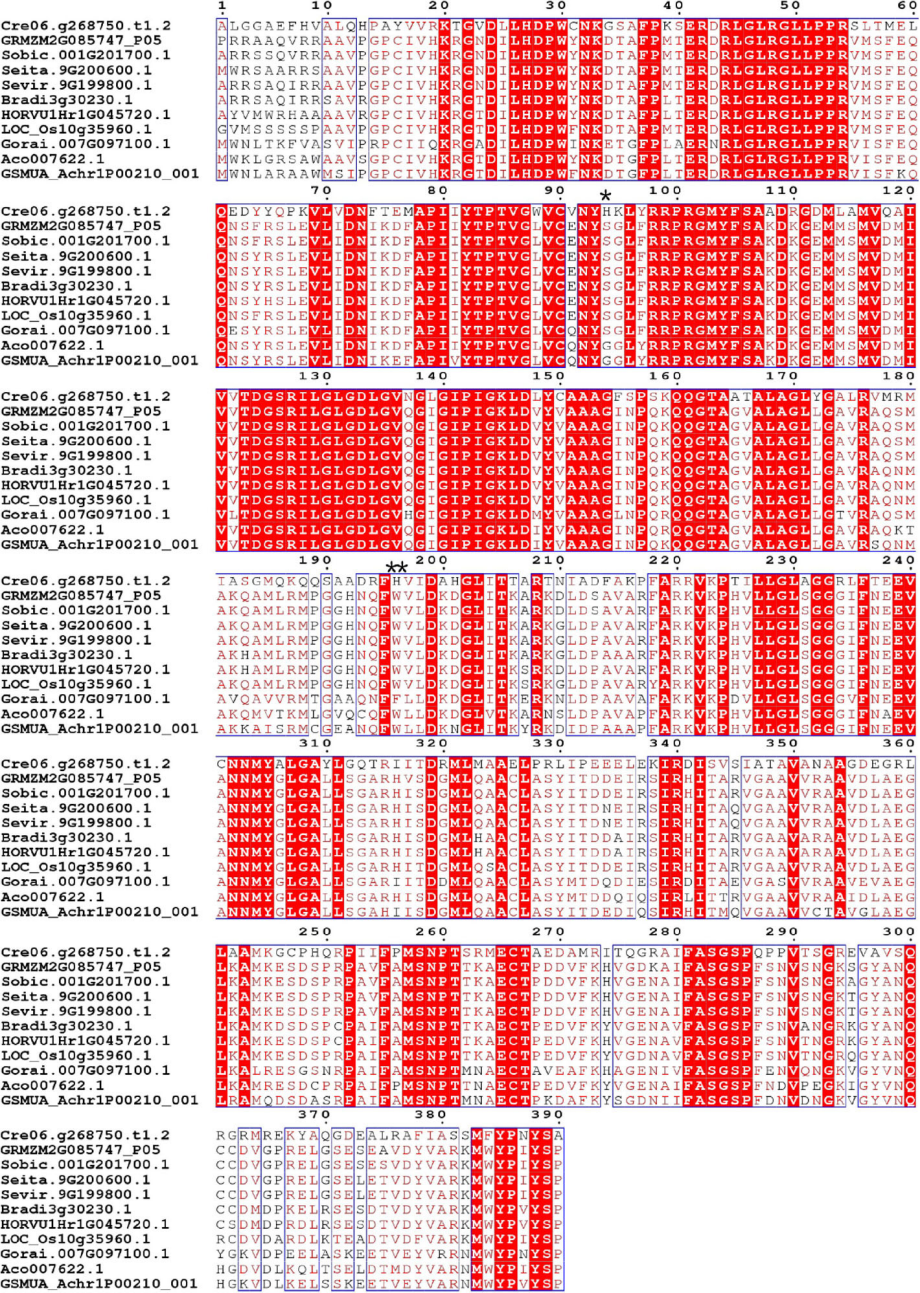
Multi-alignment of the amino acid sequences of NADP-ME in the a5 branch. *Cre06.g268750.t1.2*, *GRMZM2G085747_P05*, *Sobic.001G201700.1*, *Seita.9G200600.1*, *Sevir.9G199800.1*, *Bradi3g30230.1*, *HORVU1Hr1G045720.1*, *LOC_Os10g35960.1*, *Gorai.007G097100.1*, *Aco007622.1*, and *GSMUA_Achr1P00210_001* represent *NADP-ME* genes of *Chlamydomonas reinhardtii*, *Zea mays*, *Sorghum bicolor*, *Setaria italica*, *Setaria viridis*, *Brachypodium distachyon*, *Hordeum vulgare*, *Oryza sativa*, *Gossypium raimondii*, *Ananas comosus*, and *Musa acuminata*, respectively. Positively selected sites for NADP-ME in the above 11 monocotyledons were marked and displayed through espript3.0 (http://espript.ibcp.fr/ESPript/cgi-bin/ESPript.cgi). *Cre06.g268750.t1.2* was used as the reference sequence. Posterior probability (*P*) are indicated: *, *P* > 95%; **, *P* > 99%. Conserved regions are boxed, highly conserved loci are in red

**Fig. 5:**
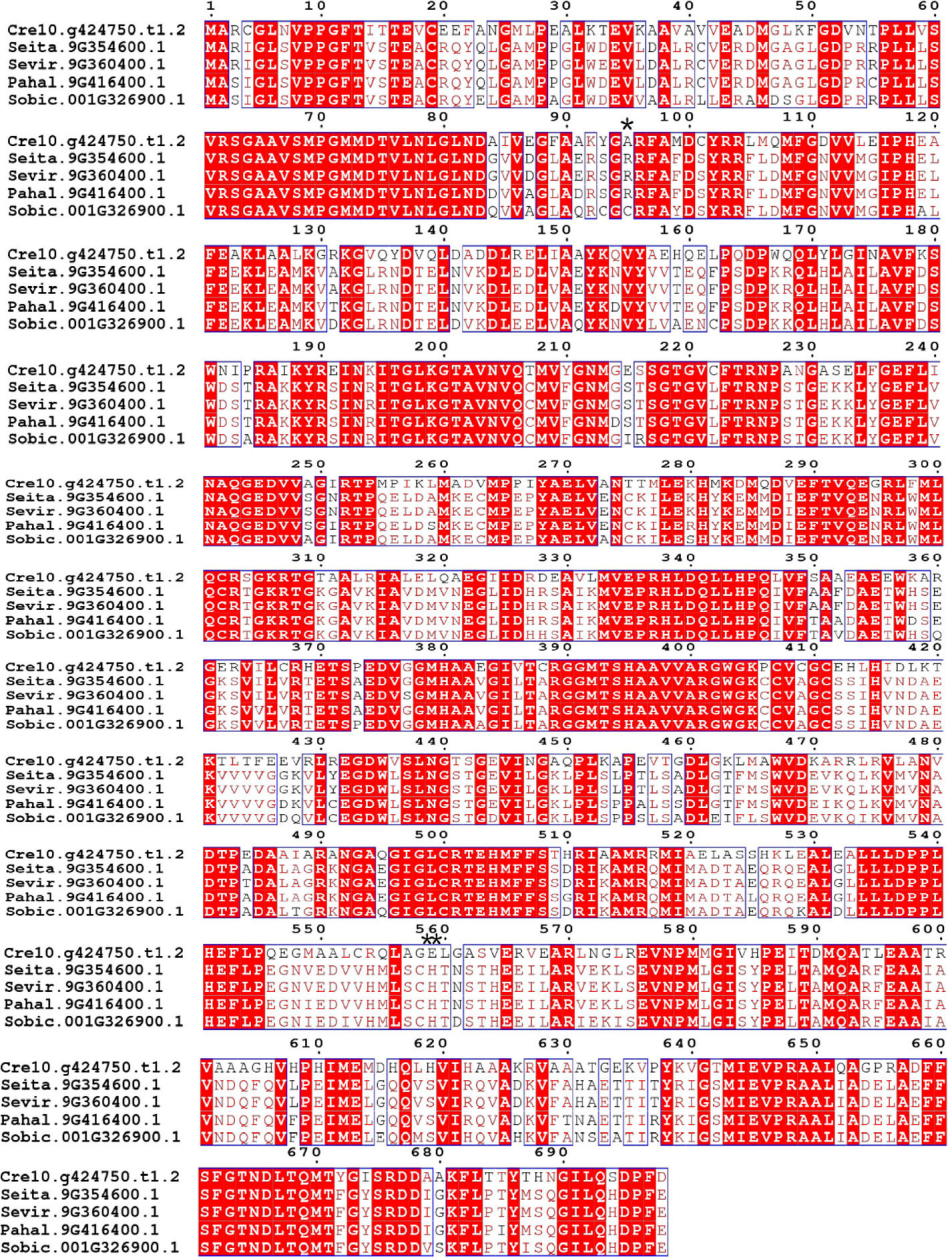
Multi-alignment of the amino acid sequences of PPDK in the e branch. *Cre10.g424750.t1.2*, *Seita.9G354600.1*, *Sevir.9G360400.1*, *Pahal.9G416400.1* and *Sobic.001G326900.1*, represent *PPDK* genes of *Chlamydomonas reinhardtii*, *Setaria italica*, *Setaria viridis*, *Panicum hallii*, and *Sorghum bicolor*, respectively. Positively selected sites for PPDK in the above five C_4_ plants were marked and displayed through espript3.0 (http://espript.ibcp.fr/ESPript/cgi-bin/ESPript.cgi). *Cre10.g424750.t1.2* was used as the reference sequence. Posterior probability (*P*) are indicated: *, *P* > 95%; **, *P* > 99%. Conserved regions are boxed, highly conserved loci are in red

Based on the above phylogenetic relationships and positive selection analysis, we conducted detailed structural and functional studies using the protein sequence alignment of NADP-ME at the a5 branch and PPDK at the e branch, which contain monocots and C_4_ plants, respectively. *Cre06.g268750.t1.2* in the a5 branch and *Cre10.g424750.t1.2* in the e branch were used as reference sequences for further analyses. Sites 94H and 196H in the a5 branch (Fig. 4) and 95A and 559E in the e branch (Fig. 5) were significantly positively selected at a posterior probability threshold of 95%. Conserved and highly conserved regions were distinguished.

### Distribution of positively selected sites on three dimensional structures of NADP-ME and PPDK

**Fig. 6:**
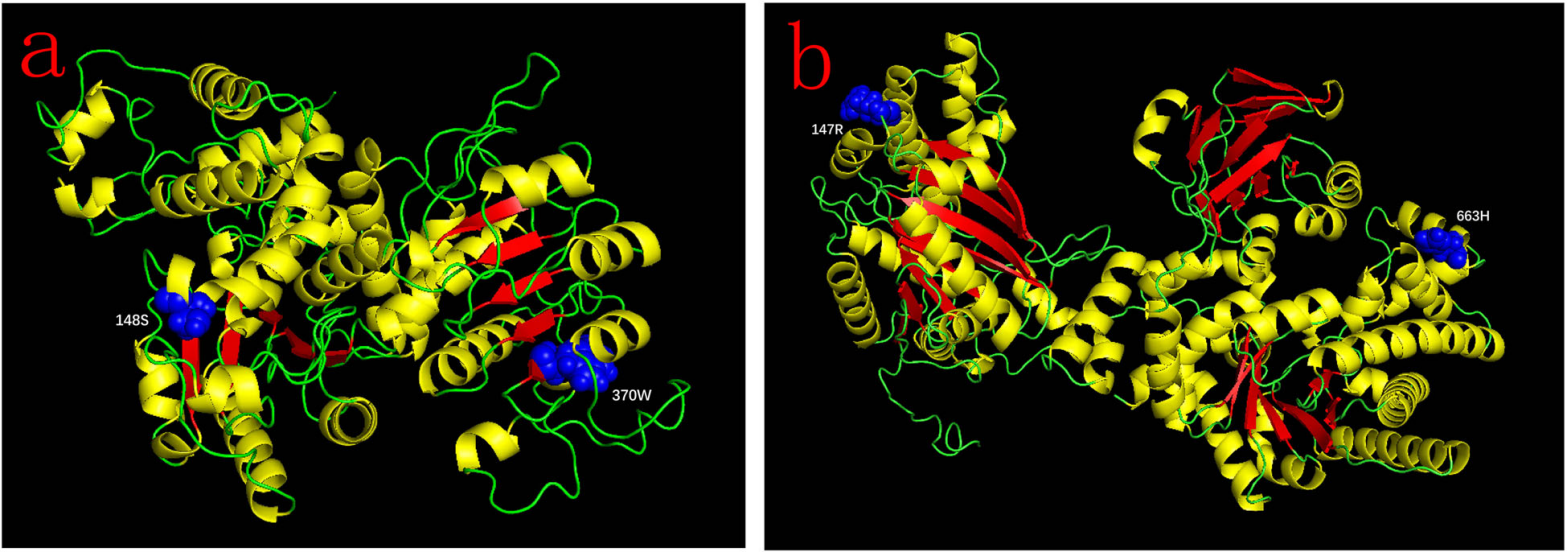
Three-dimensional models of *Setaria italica* (**a**) *seita.9G200600.1* and (**b**) *seita.9G354600.1*. Yellow color indicates the helix region, red represents sheet region, and blue corresponds to the positively selected sites. 148S, 147R and 663H was located in the helix region and 370 W was located in sheet region

We took the three-dimensional (3D) model of *seita.9G200600.1* and *seita.9G354600.1* as an example and analyzed the positively selected sites. As shown in Fig. 6a, the positively selected sites 94H and 196H in the a5 branch of NADP-ME-A were mapped to the sites 148S and 370 W of *seita.9G200600.1*. Similarly, the positively selected sites 95A and 559E in the e branch of PPDK were mapped to the sites 147R and 663H of *seita.9G354600.1* (Fig. 6b). The yellow color in 3D models indicates the helix region, red represents the sheet region, and blue corresponds to specific amino acids. 148S, 147R, 663H were located in helix regions, and 370 W was located in the sheet region (Fig. 6).

### Expression analysis of foxtail millet *NADP-ME* and *PPDK* genes determined by qRT-PCR

**Fig. 7:**
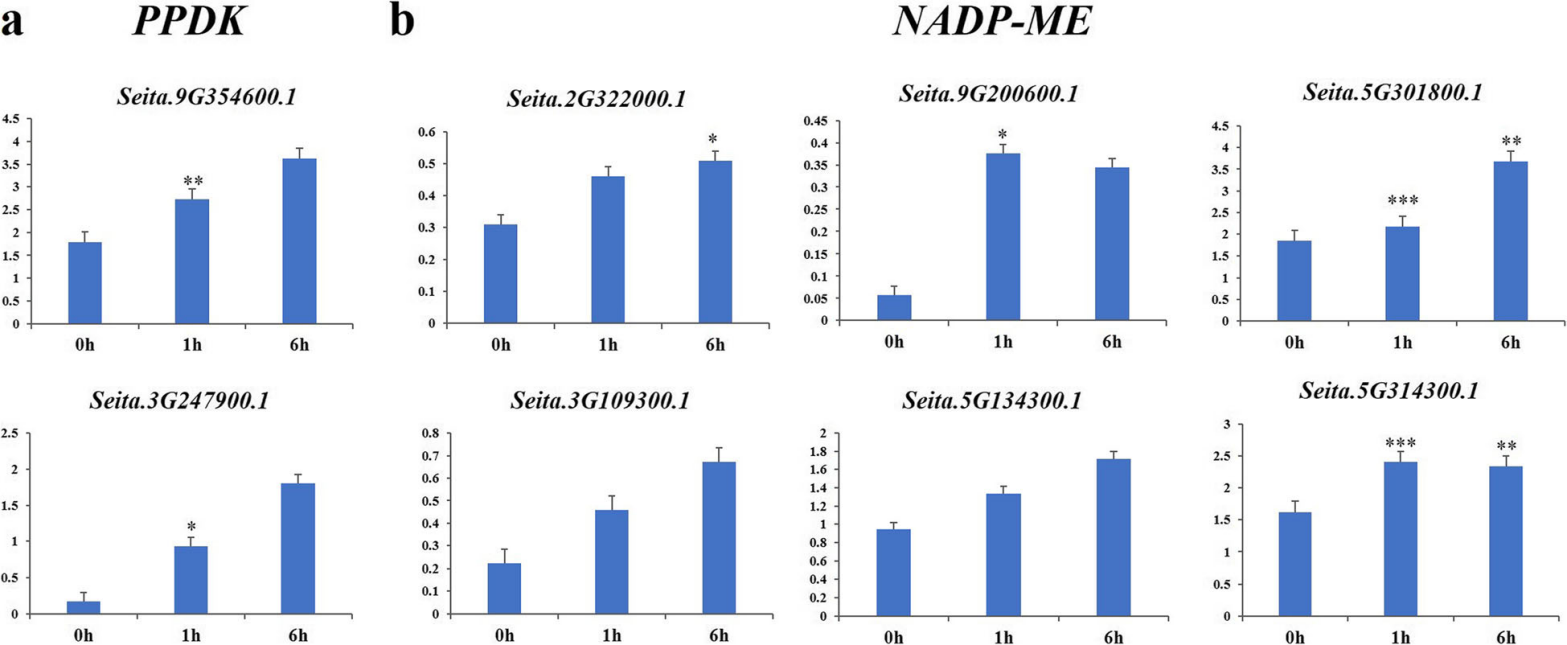
Expression profiling of (**a**) *PPDK* and (**b**) *NADP-ME* genes in foxtail millet under light treatment. Times of light exposure are shown on the x-axis

Based on the phylogenetic relationships (Figs. 1, 2 and 3), we selected 6 *NADP-ME* and 2 *PPDK* foxtail millet genes for qRT-PCR after light treatment. Expression levels of all these genes were up-regulated after light exposure for 1 h. Except for *NADP-ME* genes, *Seita.5G314300.1* and *Seita.9G200600.1*, the others had higher expression levels after light treatment for 6 h (Fig. 7; Additional file 8: Table S5).

## Discussion

### Evolution of the *NADP-ME* and *PPDK* gene families

C_4_ photosynthesis evolved approximately 30 million years ago [29]. Angiosperm C_4_ plant species then underwent 62 independent evolutionary events [30]. Most C_4_ plants are monocots, including 4600 grass and 1600 sedge species, whereas only 1600 C_4_ species from 16 families are dicots with 75% of them in families Chenopodiaceae, Amaranthaceae, Euphorbiaceae, and Asteraceae [31]. Previous research concluded that despite the specific cell structure of C_4_ plants the enzymes PEPC, NADP-ME and PPDK were essential for C_4_ photosynthesis [32, 33]. Interestingly, increases in the numbers of *NADP-ME* and *PPDK* genes occurred later in evolution. Various studies have suggested that multiple duplication events occurred during plant evolution, including the γ event that separated monocots and dicots [34], and ρ event that occurred before divergence of wheat, maize and rice, but after divergence of grasses and pineapple [35], and τ and σ events that occurred in the Poaceae [36].

In this study, 14 and 7 *NADP-ME* genes were identified in soybean and maize, respectively (Additional file 1: Table S1). Although the maize genome size (2300 Mb) is more than twice that of soybean (1100 Mb) [28, 37] the number of *NADP-ME* genes in maize is less than in soybean, indicating that expansion of the *NADP-ME* gene family was not by genome duplication, but was caused by different expansion patterns after divergence of monocot and dicot species [38, 39]. For the 35 *PPDK* genes from 25 species identified in this study most species had only one or two members (Additional file 2: Table S2). Compared to *NADP-ME* the numbers of *PPDK* genes were less but were more stable during evolution.

*NADP-ME* and *PPDK* genes are widely present in photosynthetic plant species such as algae, mosses, ferns, gymnosperms and angiosperms [40, 41]. From the phylogenetic trees constructed in this study we concluded that *NADP-ME* genes were branched into subfamilies A and B. The B2 branch containing all dicot species evolved earlier than the B1 branch containing all monocot species, suggesting that the B subfamily evolved independently after divergence of the monocots and dicots a step known as the γ event (Fig. 2) [34]. The phylogenetic tree of the *PPDK* gene family showed that monocots branched off and formed subfamily I before dicots formed subfamily II, indicating that the *PPDK* gene family evolved independently after divergence of monocots and dicots [34]. In the Poaceae there was clear clustering within monocots and dicots. For example, the *NADP* and *PPDK* genes of C_4_ plants were more closely related to each other than to C_3_ plants (Figs. 1, 2 and 3). We inferred that both the *NADP-ME* and *PPDK* gene families in the Poaceae underwent independent evolution after the ρ event in monocots [36]. In addition, *NADP-ME* and *PPDK* in C_4_ plants are more closely clustered than in C_3_ plant species, possibly due to the higher photosynthetic efficiency of C_4_ plants.

### Identification of positively selected sites and their function significance

This study used site, branch and branch-site models to investigate the effects of selection pressure on the *NADP-ME* and *PPDK* gene families. Both site and branch models failed to detect any positive sites, possibly negated by purifying selection and neutral drift [42, 43]. The branch-site model is most accurate and can detect rare positively selected sites on specific branches [44]. The branch-site model detected a total of 55 sites at a posterior probability of 0.6 that had undergone positive selection in the *NADP-ME* gene family (Tables 1 and 2). We found a total of 8, 5, 14, 5, 4, 2, and 2 positively selected sites for the a1-a7 branches, respectively, in subfamily A (Fig. 1; Table 1). In subfamily B we found 8, 0, 2, 0 and 5 positively selected sites for b1-b5 branches (Fig. 2; Table 2). The branch model for the *PPDK* gene family revealed that the ω values were much smaller than 1 for the five front branches, indicating strong purifying selection (Table 3). The branch-site model detected 1, 1, 0, 0 and 8 positively selected sites for branches a-e (Table 3).

Both site and branch models suggested that the *NADP-ME* and *PPDK* gene families had undergone mostly purifying selection while maintaining normal genes function. Detection of a few positively selected sites by the more accurate branch-site model demonstrated that only a few beneficial mutations had occurred during evolution in order to adjust to changing environments [45]. C_4_ plants are capable of utilizing lower amounts of CO_2_ compared to their C_3_ counterparts. This might be related to the positively selected sites found in both the *NADP-ME* and *PPDK* families in C_4_ plants.

Positive selection is the retention and spread of advantageous mutations throughout a population and has long been considered synonymous with shifts in protein function [45]. Determining the amount of positive selection has wide-ranging implications for understanding genome function and maintenance of genetic variation [46]. In this study, four positively selected sites, including 94H and 196H were identified in the a5 branch of NADP-ME and 95A and 559E in the e branch of PPDK at a posterior probability threshold of 95% (Figs. 4 and 5). Previous studies showed that minimal changes in the primary structure were responsible for the different kinetic behavior of each NADP-ME and PPDK isoform [47, 48]. To clarify the roles of positively selected sites in C_4_ plant evolution and explore the relationship between positively selected sites and high photosynthetic rates in C_4_ plants, 3D models of *seita.9G200600.1* and *seita.9G354600.1* were drawn. As shown in Fig. 6, positively selected sites 148S, 147R, and 663H were located in helix regions, whereas 370 W was located in a sheet region. These positive amino acid selection sites might reflect the functional divergence in C_4_ and C_3_ plants that caused C_4_ plants to possess higher photosynthetic capacity. These results also indicated that the amino acid sites of NADP-ME and PPDK family members changed during plant evolution, and that the evolutionary rates were different. It also provided a priority basis for further analysis of the functions of NADP-ME and PPDK.

Further analysis of genes in the a5 branch of NADP-ME and e branch of PPDK showed that the C_4_ plants in the a5 branch include *GRMZM2G085747_P05*, *Sobic.001G201700.1*, *Sevir.9G198800.1*, *Pahal.9G197100.1*, *Aco007622.1,* and *Seita.9G200600.1* (Fig. 1). Previous study showed that maize *GRMZM2G085747* was involved in the Calvin cycle by carbon fixation in the sheath cells of leaf vascular bundles maize (a C_4_ species) during photosynthesis [49]. Sorghum *NADP-ME* gene *Sobic.001G201700* showed high transcript abundance in the C_4_ pathway [50]. Furthermore, a comparison of one C_3_ and 11 C_4_ grass species (Poaceae) showed that the transcript abundance of *Sobic.001G201700* was consistently elevated in C_4_ species [24]. The e branch of PPDK members all belonged to C_4_ plants, including *Seita.9G354600.1*, *Sevir.9G360400.1*, *Pahal.9G416400.1,* and *Sobic.001G326900.1* (Fig. 3). A previous study reported that *Sobic.001G326900* showed a high transcript abundance in the C_4_ pathway [50]. In this study, the sites 94H and 196H in the a5 branch of NADP-ME and 95A and 559E in the e branch of PPDK were identified as positively selected at posterior probability thresholds of 95% (Figs. 4 and 5). *GRMZM2G085747* and *Sobic.001G201700* in the a5 branch of NADP-ME, and *Sobic.001G326900* in the e branch of PPDK were all involved in C_4_ photosynthesis [24, 49, 50]. Our results suggested that these sites were positively selected for high photosynthetic rates during C_4_ evolution.

## Conclusions

One hundred and sixty two *NADP-ME* and 35 *PPDK* genes characterized in 25 species had highly similar motif compositions within subfamilies. Phylogenetic analysis showed that the *NADP-ME* and *PPDK* genes can be placed in four and two branches, respectively. The *NADP-ME* and *PPDK* genes in C_4_ species had closer evolutionary relationships than in C_3_ species. Analyses of selective pressure on the *NAPD-ME* and *PPDK* gene families identified four positively selected sites, including 94H and 196H in the a5 branch of NADP-ME, 95A and 559E in the e branch of PPDK at posterior probability thresholds of 95%. The positively selected sites were located in helix and sheet region. It was inferred that positive selection was driving the evolution of NADP-ME and PPDK in C_4_ species. This study contributes to an increased understanding the roles of NADP-ME and PPDK in C_3_ and C_4_ species, and provides insights into the evolutionary biology of C_4_ plants.

## Methods

### Dataset

Conserved NADP-ME and PPDK protein sequences of Arabidopsis and rice were obtained from the public databases Uniprot (https://www.uniprot.org/) and TAIR (https://www.arabidopsis.org/). All NADP-ME and PPDK protein sequences and CDS (coding sequences) of 25 species, including representatives of algal, moss, Lycopodiophyta, monocotyledon and dicotyledon species were obtained from Phytozome V12 (https://phytozome.jgi.doe.gov/pz/portal.html) and incorporated into a local database. Each sequence was compared to the NADP-ME and PPDK protein sequences from other species and those from Arabidopsis and rice using blastp with a threshold of E < 1e-5. CDD and Pfam were used to investigate whether the sequences contained conserved NADP-ME and PPDK protein structures. Incomplete protein structures were removed.

Molecular weights and isoelectrical points of NADP-ME and PPDK protein sequences were analyzed using Expasy (https://web.expasy.org/compute_pi/).

### Construction of phylogenetic trees and analysis of conserved protein sequences

Multiple comparisons of candidate NADP-ME and PPDK protein sequences were made using the software MUSCLE3.8.31 [51]. Neighbor joining (NJ) trees were constructed with the software MEGA 7.0 using the Poisson model with 1000 bootstrap replications, gaps were filled using pairwise methods, and other parameters were based on default values [52]. Maximum likelihood (ML) trees were constructed for NADP-ME and PPDK using the Bayesian Information Criterion (BIC) and 1000 bootstrap replications with the software IQ-TREE1.6.5 [53]. The optimal model of the ML trees was estimated using the parameter M: ONLY TEST. Visualization of the constructed phylogenetic tree used Figtree.

Analysis of conserved protein sequences used the software MEME 2.12.0 with -nmotifs: 20, −minw: 10, maxw: 50 [54]. Other parameters were based on default values. Results were visualized using TBtools software.

### Analysis of natural selection pressure

The protein sequences of NADP-ME and PPDK from the multiple comparison analyses were determined using Muscle 3.8.31 software, the CDS and aligned protein sequences are submitted to the online tool PAL2NAL (http://www.bork.embl.de/pal2nal/) for codon alignment. Selection pressure was calculated using the software PAML4.9e, with ω < 1 indicating purifying selection, ω =1 indicating neutrality, and ω > 1 indicating positive selection [55]. Three methods were applied to calculate selection pressure: (1) site-specific models that adopt the M3 and M0 models in testing; (2) branch-specific models that compare the foreground branches to the background branches to test for positive selection; and (3) branch-site models (Model A), that tests for positively selected sites. Statistical analyses were performed using chi-squared tests.

### Positive selection in protein sequences and structure analysis

The aligned rearranged CDS and amino acids were entered into PAL2NAL (http://www.bork.embl.de/pal2nal/), a web tool for performing multiple codon alignments. Then the aligned sequences were visualized by ESPript v3 (http://espript.ibcp.fr/ESPript/cgi-bin/ESPript.cgi).

The full-length protein sequences of foxtail millet (*Setaria italica*) NADP-ME and PPDK were submitted to I-TASSER server (https://zhanglab.ccmb.med.umich.edu/I-TASSER/) to predict the 3D structure. Positively selected sites were tested at a posterior probability threshold of 95% in the branch-site model and mapped onto the surface of 3D structures by PyMol v2.3 (http://PyMOLwiki.org).

### Plant growth and harvesting

Foxtail millet cultivar Yugu 1 used for qRT-PCR was provided by Anyang Institute of Agricultural Sciences, Henan. Seeds were surface-sterilized in 0.5% NaClO for 1 min and cleaned three times with sterilized distilled water, then were plated on GM-agar media and stratified in darkness for 3 days at 4 °C. After germination, the seedlings were grown in darkness for 3 days at 27 °C and transferred to a growth chamber at 27 °C and light conditions (600 μmol m^− 2^ s^− 1^). After light treatment for 0, 1, and 6 h, leaves were collected, immediately frozen in liquid nitrogen and stored at − 80 °C for RNA isolation. All samples were biologically duplicated 3 times.

### qRT-PCR

Primers designed by Primer 3 using cDNA sequences from *Setaria italica* v2.2 (phytozome.jgi.doe.gov) are listed in Additional file 9: Table S6. qRT-PCRs were performed in triplicate and using SYBR® Green PCR Master Mix Kit (Applied Biosystems, GA, USA). Data acquisition and analyses were performed using the ABI7900 system (Applied Biosystems). Relative expression levels were determined using the 2^-ΔΔCT^ analysis method.

## Supplementary information

**Additional file 1: Table S1.** Characterization of *NADP-MEs* in 25 plant species.

**Additional file 2: Table S2.** Characterization of *PPDKs* in 25 plant species.

**Additional file 3: Table S3.** Consensus sequences of motifs 1–20 in *NADP-MEs*.

**Additional file 4: Table S4.** Consensus sequences of motifs 1–20 in *PPDKs.*

**Additional file 5: Figure S1.** Conserved protein motifs in *NADP-ME* genes of 25 plant species. Motif numbers 1–20 are displayed as different colored boxes. Sequence information for each motif is provided in Additional file 3: Table S3.

**Additional file 6: Figure S2.** Conserved protein motifs in *PPDK* genes of 25 plant species. Motif numbers 1–20 are displayed as different colored boxes. Sequence information for each motif is provided in Additional file 4: Table S4.

**Additional file 7: Figure S3.** Phylogenetic tree established for 162 *NADP-ME* genes in 25 species. A1, A2, B1 and B2 are represented by the red, pink, blue and green, respectively.

**Additional file 8: Table S5.** The raw data of qRT-PCR.

**Additional file 9: Table S6.** Primer sequences of 8 genes used for qRT-PCR validation.

## Data Availability

All data generated or analyzed during this study has been contained within the manuscript and supplementary information files.

## Abbreviations

3D: Three-dimensional
BIC: Bayesian Information Criterion
CAM: Crassulacean acid metabolism
CDS: Coding sequences
LRT: Likelihood ratio test
Lsu: Large subunit
ML: Maximum likelihood
NADP-ME: NADP-malic enzyme
NJ: Neighbor joining
PEPC: Phosphoenolpyruvate carboxylase
PPDK: Pyruvate orthophosphate dikinase
qRT-PCR: Quantitative RT-PCR
