# Supplementary figures and images for: Identification and evolution of C4 photosynthetic pathway genes in plants
Source: BMC Plant Biol. 2020 Mar 30;20:132. doi: 10.1186/s12870-020-02339-x (PMC7106689; doi:10.1186/s12870-020-02339-x)

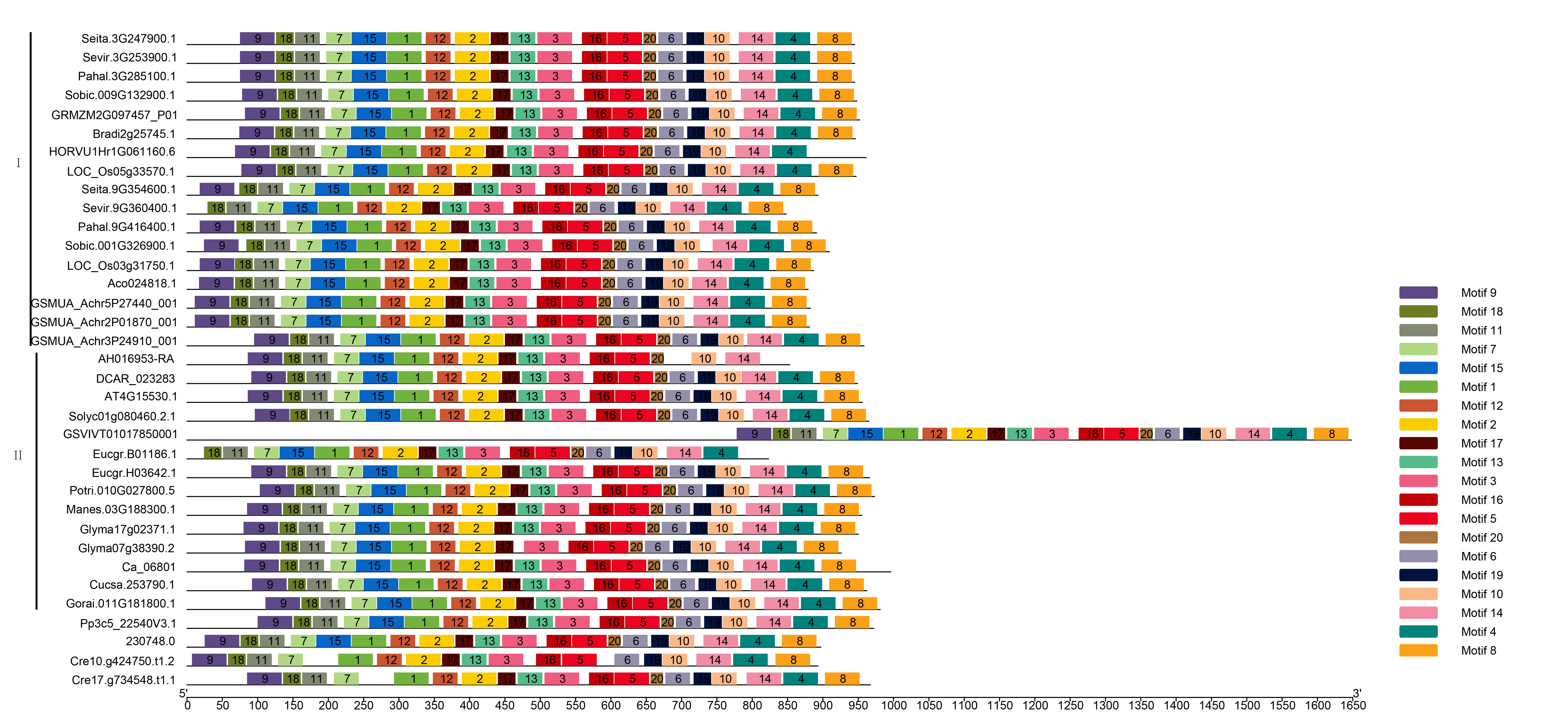

Supplement: Supplementary file 6 — Additional file 6: Figure S2. Conserved protein motifs in PPDK genes of 25 plant species. Motif numbers 1–20 are displayed as different colored boxes. Sequence information for each motif is provided in Additional file 4: Table S4. [file 12870_2020_2339_MOESM6_ESM.jpg]

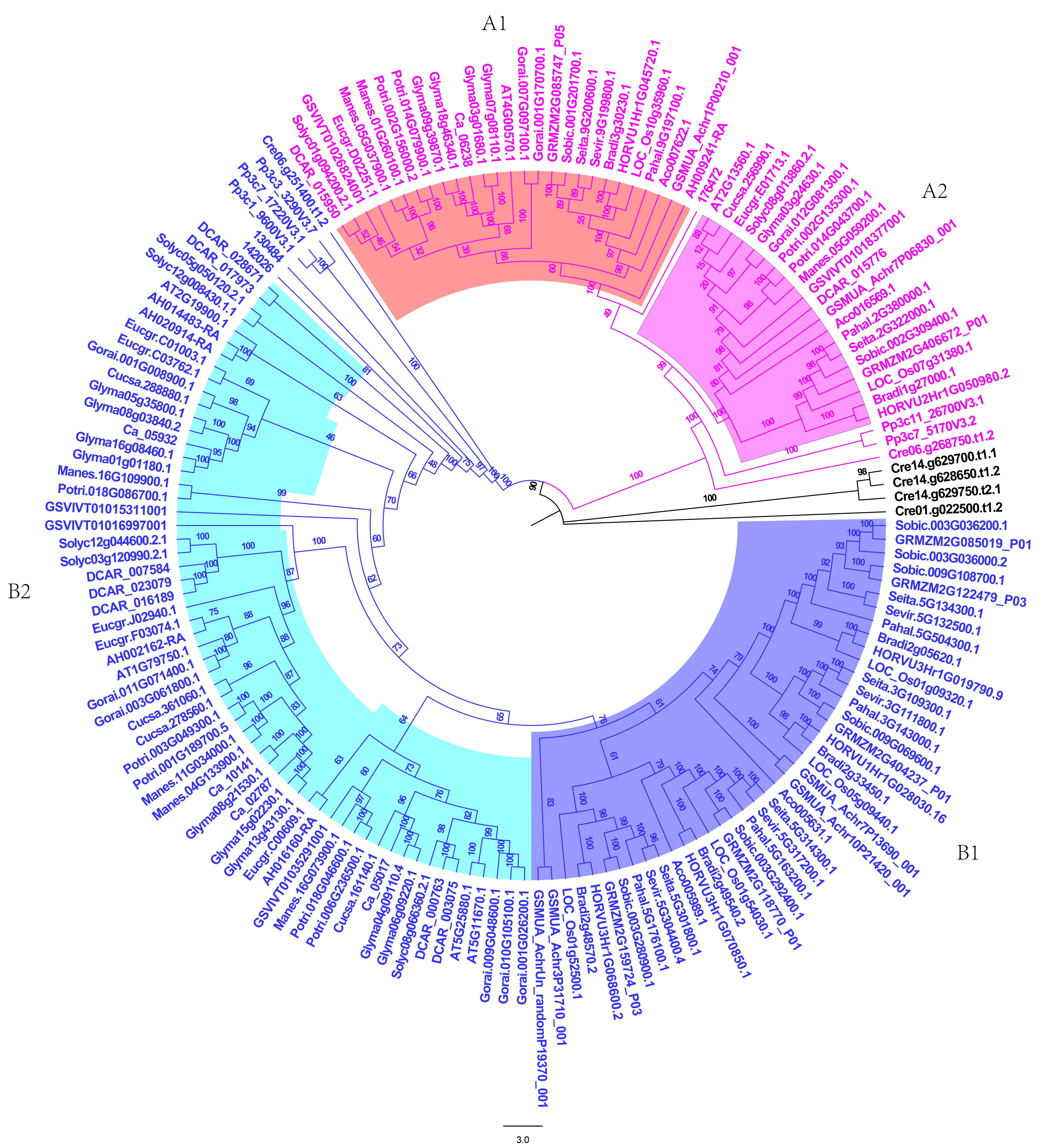

Supplement: Supplementary file 7 — Additional file 7: Figure S3. Phylogenetic tree established for 162 NADP-ME genes in 25 species. A1, A2, B1 and B2 are represented by the red, pink, blue and green, respectively. [file 12870_2020_2339_MOESM7_ESM.jpg]
